# The association between dietary sodium intake and osteoporosis

**DOI:** 10.1038/s41598-022-18830-4

**Published:** 2022-08-26

**Authors:** Susie Hong, Jong Wook Choi, Joon-Sung Park, Chang Hwa Lee

**Affiliations:** 1 Department of Internal Medicine, Hanyang University College of Medicine, 222 Wangsimni-ro, Seongdong-gu, Seoul, 04763 South Korea; 2 Research Institute of Medical Science, Konkuk University School of Medicine, Chungju, Korea

**Keywords:** Endocrinology, Nephrology

## Abstract

The association of inadequate dietary sodium intake with bone mineral density (BMD) and the risk of osteoporosis is controversial. To find the association between low sodium diet and the risk of incipient osteoporosis, we performed a population-based cross-sectional analysis using Tanaka method for estimation 24-h urinary sodium excretion (e24hUNaE_Tanaka_) as a candidate indicator of sodium intake. We identified 3869 participants without osteoporosis and classified them into quartiles according to their value of e24hUNaE_Tanaka_. BMD was measured to find participants at risk of osteoporosis. Lower e24hUNaE_Tanaka_ was related to decreasing BMD of the distal radius. Multiple Cox-proportional hazard models demonstrated that e24hUNaE_Tanaka_ had an inverse association with the risk of osteoporosis (adjusted HR = 0.859, 95% CI = 0.751–0.982) and survival analysis revealed that the lowest quartile group had poor osteoporosis-free survival (*P*_Log-rank_ < 0.0001). Furthermore, our restricted cubic spline analysis revealed that the relationship between e24hUNaE_Tanaka_ and HR of osteoporosis was negative curvilinear in males and postmenopausal females and positive linear in premenopausal females. Our findings suggest that lower sodium intake was a significant predictor of incipient osteoporosis and there was wide variation in this relationship according to sex and female hormone status.

## Introduction

Osteoporosis is one of the most common diseases in bone mineral metabolism and it can exert severe negative effects on quality of life and bone health in the aging population^1^. Furthermore, recent epidemiologic studies had shown that some osteoporotic fractures of the specific site are responsible for increased hospital morbidity and all-cause mortality risk in both inpatient and outpatient subjects^2,3^. Although osteoporosis has a multi-systemic and polygenic nature, there is very limited data to support the effect of other hidden risk factors, except age, ethnicity, and sex, on bone mineral dysmetabolism and osteoporosis^4,5^. Thus, identification of new modifiable risk factors of osteoporosis is critical to prevent its irreversible consequences.

Growing evidence showed that not only calcium but also sodium is an important molecule in regulating bone mineral metabolism^6^. However, there is wide controversy in the relationship between dietary sodium intake and the risk of osteoporosis^6^. Because previous epidemiologic studies had revealed that increased sodium intake may be related to either sodium-induced calciuria or enhancing bone reabsorption which may contribute to decreased bone mineral density (BMD), current global guidelines recommend a low salt diet to help keep our bone healthy^6–8^. Recently, some authors argued that subjects taking a low sodium diet were likely to have decreased energy intake and other important nutrients, hyponatremia, and/or activation of the renin–angiotensin–aldosterone system, which may contribute to abnormal low BMD^9,10^. Therefore, there is need for studies to elucidate the exact association between dietary sodium intake and the risk of osteoporosis.

It is well known that sex hormones influence the development of sex-specific traits and they regulate structures and functions of reproductive systems. In addition, accumulating evidence indicates that sex hormones play critical roles in maintaining bone health and that sex hormone insufficiency is strongly associated with the development of osteoporosis and bone fracture^11^. However, there is very limited data on hormonal influence on the association between a low sodium diet and the risk of incipient osteoporosis. Therefore, we conducted this population-based cross-sectional analysis using the Tanaka method for estimating 24-h urinary sodium excretion (e24hUNaE_Tanaka_) as a candidate indicator of sodium intake. After that, we performed subgroup analysis to find out the role of sex hormone on the sodium intake and osteoporosis.

## Results

### Baseline characteristics

The participants (n = 3869) comprised 1845 male, 1632 premenopausal female, and 392 postmenopausal female participants. When estimating sodium intake with the Tanaka equation, male participants tended to intake more sodium than female participants in this study (men 2.3 ± 0.5 g/day; women 1.9 ± 0.6 g/day; *P* < 0.001). Participants in the lowest quartile were older and more likely to have increased white blood cell count and platelet count. Participants in the highest quartile were more obese and had increased prevalence of diabetes mellitus, thyroid disease, higher levels of fasting glucose and more urinary excretion of albumin. Moreover, dietary energy intake increased as the 24-h urinary sodium excretion increased. The other demographic data and clinical characteristics are presented in Table 1 and Supplemental Table 1.

**Table 1:** General characteristics grouped according to e24UNaE_Tanaka_* (g/day).

	Quartile 1	Quartile 2–3	Quartile 4	
e24UNaE_Tanaka_ in males	≥ 0.8, ≤ 1.9	> 1.9, ≤ 2.6	> 2.6, ≤ 5.4	
e24UNaE_Tanaka_ in females	≥ 0.2, ≤ 1.6	> 1.6, ≤ 2.2	> 2.2, ≤ 5.3	

Variables	(n = 983)	(n = 1936)	(n = 950)	*P*
Age (year)	52.9 ± 8.9	50.8 ± 8.5	50.5 ± 8.1	< 0.0001
Sex (male, %)	473 (47)	910 (48)	462 (49)	0.9985
Current smoker (n, %)	300 (31)	519 (27)	235 (25)	0.0048
Menopause (n, %)	110 (11)	193 (10)	89 (9)	0.7409
Years since menopause (year)	11.5 ± 7.8	10.6 ± 7.7	10.3 ± 7.2	0.4849
Body mass index (kg/m^2^)	24.2 ± 3.2	24.4 ± 3.0	24.5 ± 3.1	0.1745
Waist circumference (cm)	81.2 ± 8.8	82.4 ± 8.8	83.6 ± 8.9	< 0.0001
Systolic BP (mmHg)	122.8 ± 17.7	122.0 ± 17.4	124.0 ± 17.8	0.0319
Diastolic BP (mmHg)	81.2 ± 11.5	81.0 ± 11.6	82.2 ± 11.3	0.0360
**Medical history**				
Diabetes mellitus (n, %)	44 (4)	110 (6)	82 (9)	0.0008
Hypertension (n, %)	134 (14)	243 (13)	114 (12)	0.3870
Dyslipidemia (n, %)	17 (2)	50 (3)	26 (3)	0.3108
Thyroid disease (n, %)	16 (2)	68 (4)	35 (4)	0.0123
Cardiovascular disease (n, %)	13 (1)	26 (1)	19 (2)	0.3906
Chronic lung disease (n, %)	12 (1)	17 (1)	5 (1)	0.2286
Chronic liver disease (n, %)	41 (4)	89 (5)	47 (5)	0.8028
**Laboratory**				
White blood cell (10^9^/L)	6.8 ± 1.9	6.6 ± 1.8	6.6 ± 1.8	0.0363
Hemoglobin (g/dL)	13.7 ± 1.5	13.6 ± 1.6	13.5 ± 1.5	0.1440
Platelet (10^3^/μL)	269.3 ± 66.4	261.7 ± 62.0	259.6 ± 58.8	0.0019
Sodium (mmol/L)	142.4 ± 2.4	142.4 ± 2.3	142.1 ± 2.3	0.0075
Potassium (mmol/L)	4.49 ± 0.43	4.51 ± 0.42	4.51 ± 0.46	0.3905
Total protein (g/dL)	7.19 ± 0.37	7.19 ± 0.39	7.19 ± 0.40	0.9324
Albumin (g/dL)	4.08 ± 0.20	4.08 ± 0.19	4.08 ± 0.21	0.8751
Corrected calcium (g/dL)	9.71 ± 0.34	9.69 ± 0.34	9.72 ± 0.36	0.1164
Fasting blood glucose (mg/dL)	83.4 ± 17.9	84.8 ± 19.1	87.1 ± 26.0	0.0075
Post-prandial glucose (mg/dL)	123.8 ± 45.6	123.2 ± 48.7	123.2 ± 52.9	0.1082
Hemoglobin A1c (%)	5.74 ± 0.70	5.76 ± 0.88	5.84 ± 0.97	0.2294
eGFR^†^ (mL/min/1.73 m^2^)	94.4 ± 12.1	96.2 ± 12.0	97.7 ± 11.9	< 0.0001
Total bilirubin (mg/dL)	0.58 ± 0.31	0.58 ± 0.28	0.58 ± 0.33	0.3956
AST (IU/L)	30.3 ± 21.6	29.6 ± 15.1	29.3 ± 13.3	0.1588
ALT (IU/L)	27.6 ± 24.3	28.1 ± 22.1	28.0 ± 18.6	0.3352
γ- Glutamyl transferase (IU/L)	33.3 ± 51.9	35.0 ± 74.4	33.1 ± 46.0	0.9013
Triglyceride (mg/dL)	159.8 ± 88.3	159.3 ± 97.5	171.5 ± 125.5	0.0667
HDL-cholesterol (mg/dL)	43.3 ± 9.8	44.3 ± 9.8	43.8 ± 9.8	0.0201
LDL-cholesterol (mg/dL)	109.2 ± 29.4	109.2 ± 29.8	104.0 ± 31.2	< 0.0001
C-reactive protein (mg/dL)	0.26 ± 0.50	0.19 ± 0.26	0.20 ± 0.42	< 0.0001
UACR (mg/g Cr)	9.3 ± 6.7	10.0 ± 6.3	12.2 ± 7.5	< 0.0001
**Daily intake**				
Dietary energy intake (Kcal/day)	1988 ± 807	2051 ± 826	2089 ± 796	0.0029
Dietary Na intake (g/day)	3.24 ± 1.78	3.37 ± 1.91	3.45 ± 1.74	0.0044
Dietary K intake (g/day)	2.66 ± 1.39	2.79 ± 1.43	2.80 ± 1.32	0.0133
Dietary Ca intake (g/day)	0.48 ± 0.28	0.52 ± 0.31	0.52 ± 0.29	0.0049
Daily alcohol intake (g/day)	18.2 ± 26.1	19.7 ± 27.3	24.2 ± 34.2	0.0134
**Fasting morning urine sample**				
e24UNaE_Tanaka_ (g/day)	1.45 ± 0.33	2.08 ± 0.26	2.78 ± 0.42	< 0.0001
FE_Na_ (%)	0.5 ± 0.3	0.8 ± 0.3	1.4 ± 0.8	< 0.0001
UKCR (mmol/mmol)	5.4 ± 3.5	5.8 ± 3.2	7.9 ± 6.8	< 0.0001
UNaKR (mmol/mmol)	2.3 ± 1.3	3.4 ± 1.6	4.4 ± 2.0	< 0.0001
UCaCR (mg/dL/mg/dL)	0.11 ± 0.08	0.28 ± 6.68	0.16 ± 0.09	< 0.0001
FE_Ca_ (%)	0.9 ± 0.6	2.1 ± 0.5	1.3 ± 0.7	< 0.0001
**Bone mineral densitometry**				
SoS of DR at base (m/s)	4222 ± 164	4227 ± 159	4221 ± 149	0.6611
ΔSoS of DR (m/s/year)	−0.89 ± 1.31	−0.77 ± 1.18	−0.75 ± 1.19	0.0277
T-score of DR at base	0.59 ± 1.33	0.63 ± 1.28	0.58 ± 1.22	0.7428
ΔT-score of DR (/year)	−0.30 ± 0.45	−0.26 ± 0.42	−0.25 ± 0.42	0.0385
SoS of MT at base (m/s)	3946 ± 152	3968 ± 132	3967 ± 124	0.0009
ΔSoS of MT (m/s/year)	−0.78 ± 2.19	−0.82 ± 1.19	−0.69 ± 1.18	0.0650
T-score of MT at base	−0.04 ± 1.30	0.15 ± 1.23	0.14 ± 1.17	0.0012
ΔT-score of MT (/year)	−0.30 ± 0.46	−0.29 ± 0.45	−0.26 ± 0.43	0.1686
Osteoporosis progression^ǂ^ (n,%)	238 (24)	402 (21)	181 n	0.0051

Results are expressed as mean ± SD or frequencies (and proportions).

e24UNaE, estimated 24-h urine sodium excretion; BP, blood pressure; eGFR, estimated glomerular filtration rate; AST, Aspartate aminotransferase; ALT, Alanine aminotransferase; HDL, high-density lipoprotein; LDL, low-density lipoprotein; UACR, Urine albumin/Cr ratio; Cr, creatinine, Na, sodium; K, potassium; Ca, calcium; FE_Na_, fractional excretion of sodium; UKCR, urine potassium/creatinine ratio; UNaKR, urine sodium/potassium ratio; FE_Ca_, fractional excretion of calcium; SoS, speed of sound; DR, distal radius; MT, midshaft tibia.

*e24UNaE calculated using Tanaka method.

^†^Estimated using the Chronic Kidney Disease Epidemiology Collaboration equation.

^ǂ^Defined as a bone mineral density T-score at ether distal radius or midshaft of tibia below −2.5.

### Estimated 24-h dietary sodium intake and bone densitometry

**Figure 1:**
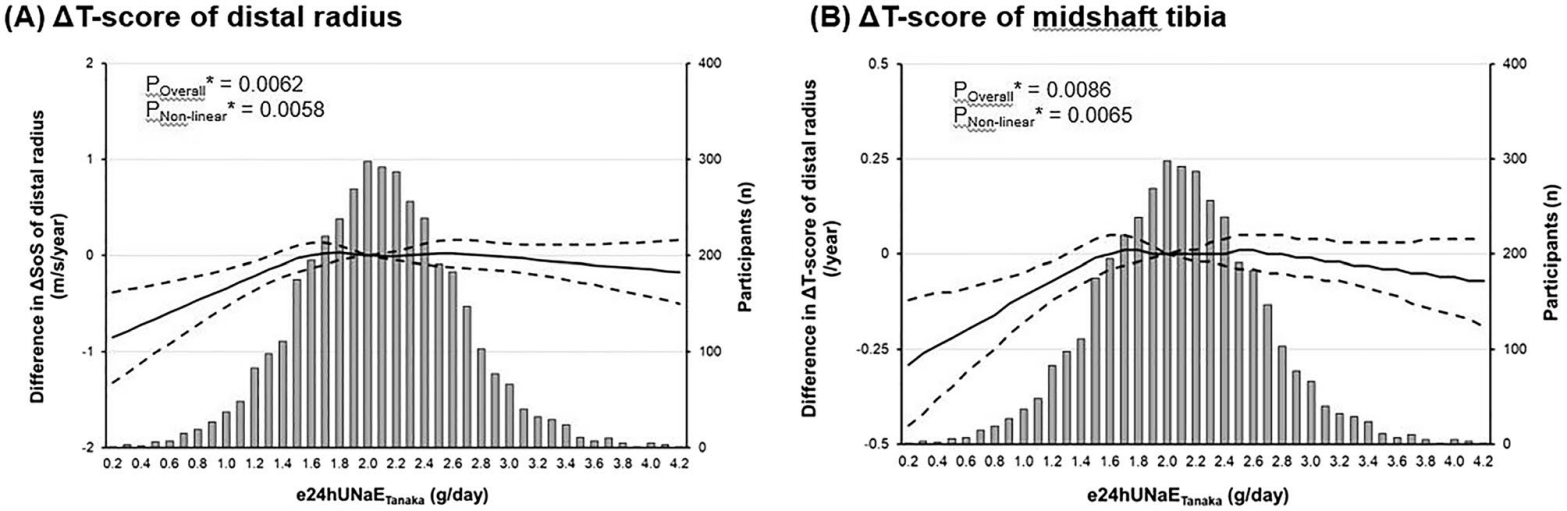
Non-linear relation of e24UNaE_Tanaka_ with the change of (**A**) ΔT-score of distal radius and (**B**) ΔT-score of midshaft tibia compared with the chosen reference e24UNaE_Tanaka_ of 2.0 g/day. Solid line represents the change of bone marrow densitometry indicators and dash lines 95% confidential intervals. *Calculated by restricted cubic spline regression using age, sex, and smoking history as covariates.

We performed linear regression analysis with age, sex, and smoking history as covariates to find the possible relation of e24UNaE_Tanaka_ with other baseline characteristics related to osteoporosis. As shown in the Table 2, we found that e24UNaE_Tanaka_ was strongly associated with clinical parameters of systemic inflammation, such as C-reactive protein, hemoglobin, platelet, and albumin, but marginally related with change of areal BMD. Unfortunately, in subgroup analysis according to sex, we did not find a significant relation between e24UNaE_Tanaka_ and the change of areal BMD during the study period (Fig. 1 and Supplemental Table 2).

**Table 2:** Linear regression for e24UNaE_Tanaka_ (g/day).

Variable	Crude		Model I	
	Slope	*P*	Slope	*P*
Age (year)	−0.0055	< 0.0001		
Sex (vs. male)	−0.4168	< 0.0001		
Current smoker (vs. non-smoker)	−0.1349	< 0.0001		
Menopause (vs. pre-menopause)	−0.0303	0.5103		
Years since menopause (year)	−0.0055	0.0902		
Body mass index (kg/m^2^)	0.0036	0.2270		
Waist circumference (cm)	0.0110	< 0.0001	0.0076	< 0.0001
Systolic BP (mmHg)	0.0034	< 0.0001	0.0020	< 0.0001
Diastolic BP (mmHg)	0.0057	< 0.0001	0.0025	0.0013
**Laboratory**				
White blood cell (10^9^/L)	−0.0033	0.5113		
Hemoglobin (g/dL)	−0.0798	< 0.0001	−0.0250	0.0014
Platelet (10^3^/μL)	−0.0010	< 0.0001	−0.0006	< 0.0001
Sodium (mmol/L)	−0.0005	0.9059		
Potassium (mmol/L)	0.0984	< 0.0001	0.0263	0.1949
Total protein (g/dL)	−0.0127	0.5790		
Albumin (g/dL)	−0.1622	0.0006	−0.0868	0.0547
Corrected calcium (g/dL)	0.1444	< 0.0001	0.0409	0.1145
Fasting blood glucose (mg/dL)	0.0023	< 0.0001	0.0015	< 0.0001
Post-prandial glucose (mg/dL)	−0.0001	0.5023		
Hemoglobin A1c (%)	0.0387	0.0001	0.0379	0.0004
eGFR* (mL/min/1.73 m^2^)	0.0049	< 0.0001	0.0037	< 0.0001
Total bilirubin (mg/dL)	−0.0844	0.0067	−0.0721	
AST (IU/L)	−0.0019	0.0009	−0.0002	0.6636
ALT (IU/L)	−0.0025	< 0.0001	−0.0003	0.5220
γ- Glutamyl transferase (IU/L)	−0.0009	< 0.0001	−0.0001	0.4669
Triglyceride (mg/dL)	0.0006	< 0.0001	0.0003	0.0004
HDL-cholesterol (mg/dL)	−0.0027	0.0051	−0.0005	0.6020
LDL-cholesterol (mg/dL)	−0.0018	< 0.0001	−0.0012	0.0004
C-reactive protein (mg/dL)	−0.0757	0.0011	−0.0504	0.0442
UACR (mg/g Cr)	0.0115	< 0.0001	0.0051	0.0006
**Daily intake**				
Dietary energy intake (Kcal/day)	0.0001	< 0.0001	0.0001	< 0.0001
Dietary Na intake (g/day)	0.0001	< 0.0001	0.0001	0.0002
Dietary K intake (g/day)	0.0001	0.0107	0.0001	0.0082
Dietary Ca intake (g/day)	0.0001	0.0115	0.0001	0.0026
Daily alcohol intake (g/day)	0.0030	< 0.0001	0.0013	0.0056
**Fasting morning urine sample**				
FE_Na_ (%)	0.6611	< 0.0001	0.6329	< 0.0001
UKCR (mmol/mmol)	0.0413	< 0.0001	0.0327	< 0.0001
UNaKR (mmol/mmol)	0.1268	< 0.0001	0.1219	< 0.0001
UCaCR (mg/dL/mg/dL)	0.0013	0.5145		
FE_Ca_ (%)	0.0002	0.4654		
**Bone mineral densitometry**				
SOS of DR at base (m/s)	0.0004	< 0.0001	0.0001	0.2067
ΔSOS of DR (m/s/year)	0.0277	0.0031	0.0124	0.1584
T-score of DR at base	−0.0189	0.0149	−0.0095	0.1912
ΔT-score of DR (/year)	0.0830	0.0019	0.0295	0.2404
SOS of MT at base (m/s)	0.0005	< 0.0001	0.0002	0.0226
ΔSOS of MT (m/s/year)	0.0196	0.0100	0.0049	0.4970
T-score of MT at base	0.0536	< 0.0001	0.0160	0.0376
ΔT-score of MT (/year)	0.0747	0.0032	0.0388	0.1030

Model I, adjusted for age, sex, and smoking history.

### Dietary salt intake and osteoporosis

**Figure 2:**
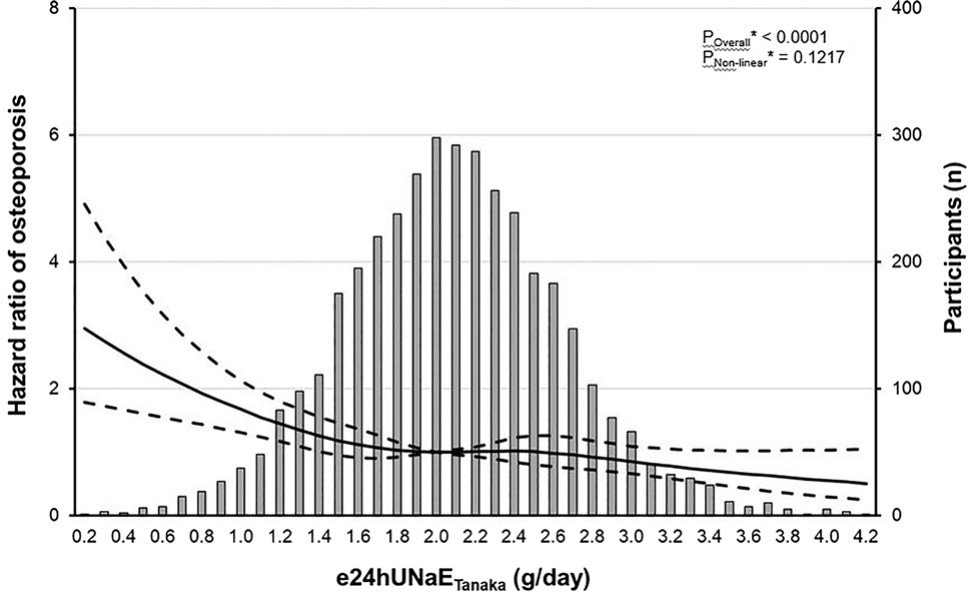
Relationship between dietary salt intake and hazard ratio of osteoporosis*. Solid line represents the adjusted HR** of incipient osteoporosis and dash lines 95% CIs**. *Defined as a bone mineral density T-score at either distal radius or tibia shaft below −2.5. **Calculated by restricted cubic spline Cox-proportional hazard regression model using age, sex, and smoking history as covariates and body mass index, waist circumference, systolic BP, diastolic BP, hemoglobin, sodium, corrected calcium, hemoglobin A1c, eGFR, triglyceride, LDL-cholesterol, UACR, and urine potassium/creatinine ratio as predictors. e24hUNaE_Tanaka_, Tanaka method for estimating 24-h urinary sodium excretion.

We performed Cox proportional-hazard model, using age, sex, and smoking history as covariates, to find an independent predictor of incipient osteoporosis. We found that decreased dietary salt intake was significantly associated with the development of osteoporosis and this association was not attenuated by further adjustment for BMI, WC, hemoglobin, corrected calcium, eGFR, and TG (e24UNaE_Tanaka_, adjusted HR = 0.859, 95% CI = 0.751–0.982, Table 3). To evaluate the linearity between e24UNaE_Tanaka_ and the risk of osteoporosis, we performed multiple adjusted RCS analyses and found that there was a negative sublinear relationship between them in entire participants (Fig. 2).

**Table 3:** Cox proportional-hazard model for osteoporosis*.

Variable	Model I		Model II		Model III	
	OR	95% CI	OR	95% CI	OR	95% CI
Menopause (vs. pre-menopause)	1.040	0.828–1.305				
Years since menopause (year)	1.001	0.979–1.025				
Body mass index (kg/m^2^)	1.036	1.020–1.053				
Waist circumference (cm)	1.016	1.010–1.022				
Systolic BP (mmHg)	1.008	1.005–1.010				
Diastolic BP (mmHg)	1.008	1.004–1.013				
**Medical history**						
Diabetes mellitus	1.103	0.903–1.348	1.112	0.899–1.375		
Hypertension	1.229	1.075–1.405				
Dyslipidemia	1.006	0.696–1.454				
Thyroid disease	1.193	0.893–1.592				
Cardiovascular disease	1.160	0.821–1.640				
Chronic lung disease	1.001	0.995–1.007				
Chronic liver disease	1.028	0.789–1.341				
**Laboratory**						
White blood cell (10^9^/L)	1.008	0.979–1.038				
Hemoglobin (g/dL)	1.048	1.002–1.096				
Platelet (10^3^/μL)	1.000	0.999–1.001				
Sodium (mmol/L)	1.025	1.001–1.049				
Potassium (mmol/L)	0.996	0.877–1.131				
Total protein (g/dL)	0.976	0.885–1.075				
Albumin (g/dL)	0.982	0.892–1.082				
Corrected calcium (g/dL)	1.315	1.169–1.479				
eGFR (mL/min/1.73 m^2^)	0.987	0.896–1.087				
Fasting blood glucose (mg/dL)	0.998	0.995–1.001				
Post-prandial glucose (mg/dL)	1.000	0.999–1.001				
Hemoglobin A1c (%)	1.081	1.029–1.137				
Total bilirubin (mg/dL)	0.805	0.646–1.002				
AST (IU/L)	1.000	0.996–1.003				
ALT (IU/L)	1.000	0.998–1.002				
γ- Glutamyl transferase (IU/L)	1.000	0.999–1.001				
Triglyceride (mg/dL)	1.001	1.001–1.001				
HDL-cholesterol (mg/dL)	0.998	0.993–1.004				
LDL-cholesterol (mg/dL)	1.004	1.001–1.011				
C-reactive protein (mg/dL)	1.046	0.997–1.120				
UACR (mg/g Cr)	1.017	1.008–1.027				
**Daily intake**						
Dietary energy intake (Kcal/day)	1.000	0.999–1.001				
Dietary Na intake (g/day)	1.000	0.999–1.001				
Dietary K intake (g/day)	0.984	0.936–1.035				
Dietary Ca intake (g/day)	0.985	0.779–1.245				
Daily alcohol intake (g/day)	0.999	0.995–1.003				
**Fasting morning urine sample**						
e24UNaE_Tanaka_ (g/day)	0.736	0.636–0.852	0.792	0.685–0.916	0.859	0.751–0.982
FE_Na_ (%)	1.140	1.024–1.270	1.055	0.924–1.204		
UKCR (mmol/mmol)	1.023	1.012–1.034	1.024	1.011–1.037		
UNaKR (mmol/mmol)	0.961	0.924–1.001				
UCaCR (mg/dL/mg/dL)	0.991	0.941–1.043				
FE_Ca_ (%)	0.999	0.990–1.007				

*Defined as a bone mineral density T-score at distal radius or tibia shaft below −2.5.

Model I, performed using age, sex, and smoking history as covariates.

Model II, performed using age, sex, and smoking history as covariates and body mass index, waist circumference, systolic BP, diastolic BP, hemoglobin, sodium, corrected calcium, hemoglobin A1c, triglyceride, LDL-cholesterol, and UACR as predictors.

Model III, performed using age, sex, and smoking history as covariates and body mass index, waist circumference, systolic BP, diastolic BP, hemoglobin, sodium, corrected calcium, hemoglobin A1c, triglyceride, LDL-cholesterol, UACR, and UKCR as predictors.

HR, hazard ratio; CI, confidence interval.

**Figure 3:**
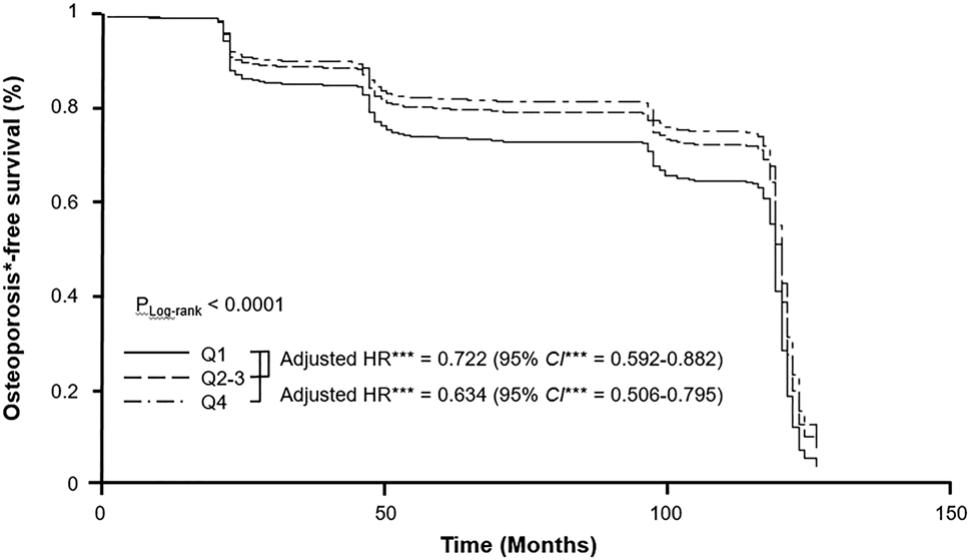
The impact of dietary salt intake on osteoporosis-free survival. Q1 had a poor osteoporosis-free survival rate compared with other groups, but there was no significant difference in adjusted HR between Q2–3 and Q4 (adjusted HR* = 0.878, 95% CI* = 0.735–1.050). *Calculated by Cox-proportional hazard model using age, sex, and smoking history as covariates and body mass index, waist circumference, systolic BP, diastolic BP, hemoglobin, sodium, corrected calcium, hemoglobin A1c, eGFR, triglyceride, LDL-cholesterol, UACR, and urine potassium/creatinine ratio as predictors. HR, hazard ratio; CI, confidence interval.

Subsequent Kaplan–Meier analysis with multiple Cox-proportional hazard regression models and log-rank test was performed to compare osteoporosis-free survival among the groups (Fig. 3). We found that participants with the lowest e24UNaE_Tanaka_ quartile had worse osteoporosis-free survival rate.

### Sex disparity in the association between dietary salt intake and osteoporosis

**Figure 4:**
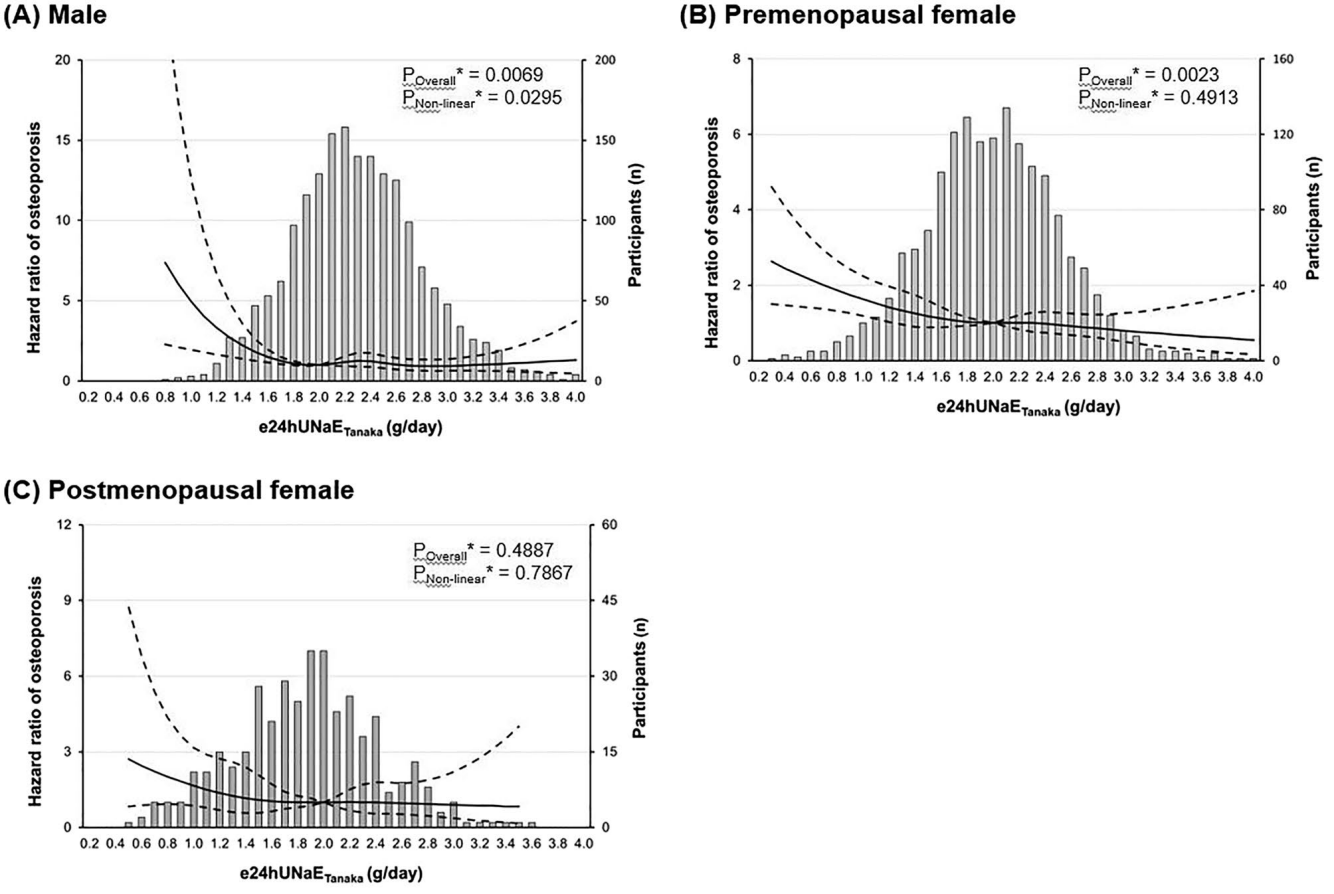
Sex disparity in the relationship between dietary salt intake and hazard ratio of osteoporosis. Solid line represents the adjusted HR* of osteoporosis and dash lines 95% CIs*. *Calculated by restricted cubic spline Cox-proportional hazard regression model using age, sex, and smoking history as covariates and body mass index, waist circumference, hemoglobin, corrected calcium, estimated glomerular filtration rate, triglyceride, and urine potassium/creatinine ratio as predictor.

There was inconsistency between our linear regression and Cox-proportional hazard model results. (Supplemental Table 3) To explore possible confounding factor(s) that could influence the relationship between dietary sodium intake and the risk of incipient osteoporosis, we classified all participants according to their sex and female menopausal status and performed further subgroup analyses. As shown in Fig. 4, our RCS analysis result revealed that there was wide variation in the association between dietary sodium intake and the risk of osteoporosis according to sex hormone status. There was a negative curvilinear relationship in male participants and postmenopausal female participants. In addition, there was a positive linear relationship in premenopausal female participants.

## Discussion

This study provided comprehensive information on the effect of dietary sodium intake on the risk of incipient osteoporosis in the general population. Our results demonstrated that e24UNaE_Tanaka_ were independent predictors of developing incipient osteoporosis. Tanaka method was developed in Asian participants^12^. It has the least amount of bias among Kawasaki method and Mage method^13^. Furthermore, there was wide variation in this association according to sex and female sex hormone state.

Growing evidence showed that osteoporosis and related sequelae have a considerable impact on health-related quality of life including fracture and subsequent morbidity. Particularly, osteoporosis is strongly associated with increased cardiovascular morbidity and mortality in the elderly population^1,3,14,15^. Although various anti-osteoporosis medications are frequently prescribed to prevent unwanted consequences, it is true that there are potential limitations in the wide use of these medical treatments^16^. Recently, some authors argued that several nutritional strategies could be another choice to reduce the risk of bone loss and fracture^17–20^. Previous epidemiologic studies had demonstrated that excess sodium intake was a potent risk of arterial hypertension and cardiovascular disease and may be associated with decreased urinary calcium reabsorption and increased bone resorption^6–8^. Accordingly, current guidelines recommended that adults at risk of chronic disease should take a low sodium diet in terms of improving body health^21–24^. However, the effect of modulation of dietary sodium intake on BMD remains controversial. In our study, we found that there was an inverse association between the estimated sodium intake per day and the risk of osteoporosis in the whole population. It is known that low sodium diet activates the renin-angiotensin-aldostereone system (RAAS) and the components of RAAS are discovered in bone tissue^25^. When RAAS is activated, it stimulates osteoclast formation and inhibits osteoblast activity to cause osteoporosis^26,27^. Moreover, insufficient sodium intake leads to a lack of other important nutrients^9,10^. A low salt diet increases the risk of osteoporosis by consuming fewer calories and increasing bone resorption markers^28^. Such findings suggested that a balanced nutrition diet is critical to bone health.

The novel finding of our study was that the effect of dietary sodium intake on the risk of incipient osteoporosis varied by sex and menopausal state. Such a result may imply that other confounding factors caused by changes in sex hormones can modulate the effect of sodium intake on bone health^6,23^. Sex hormones play a critical role in the growth and maintenance of the skeletal system. Manolagas, et. al. demonstrated that estrogen affects several cell types to attenuate bone resorption^29^.Narla, et. al. revealed that estrogen regulates mainly cortical bone turnover, but testosterone effects on the trabecular bone^11^. However, there is very limited clinical evidence on the interaction effects of abnormal dietary habits and sex hormonal status on bone homeostasis. In this regard, our results confirmed that sex hormones could regulate the effect of a low sodium diet on the onset of early osteoporosis.

In this study, there was wide inconsistency between results of linear regression analysis showed complex relation between dietary sodium intake and the change of BMD and those of Cox-proportional hazard model revealed apparent negative effects of dietary sodium intake on the risk of osteoporosis. The possible explanations are as following: (i) BMD changes over a long-time scale^30^; (ii) delayed epidemic of incipient osteoporosis was frequently observed in senile population^31,32^; and (iii) KoGES was designed to find potential risk factor(s) of chronic disease development in local population group after middle age. It may be another possible explanation that osteoporosis is a polygenic disorder and unknown confounding variables can change the impact of dietary sodium intake on BMD.

Our RSC analysis demonstrated that the osteoporotic effect of a low sodium diet was more prominent in the distal radius rather than the midshaft tibia. It was consistent with a previous prospective cohort study showing that the effect of dietary modulation on the BMD change varied according to the skeletal sites, in which increased intake of dietary sodium reduced the risk of incipient fracture at only femur neck, but not other fracture sites^23^. However, there is very little experimental evidence to explain these findings. Thus, further studies are needed to evaluate the site-specificity of the osteoporotic effect of decreased sodium intake.

There were several limitations in our study. First, this population-based study did not include data about sex hormone levels in serum, 24-h urine collection of sodium, dual-energy x-ray absorptiometry, and bone markers, such as parathyroid hormone, alkaline phosphatase, osteocalcin or c-telopeptide. Because of these limitations of study design, control of some confounding factors was not possible and a more precise analysis between sex hormone state, dietary sodium intake, BMD change, and the risk of osteoporosis was not performed. In addition, since the prevalence and severity of osteoporosis are influenced by a wide variety of factors, we could not adjust for many other factors other than age, sex, smoking history, body mass index, waist circumference, hemoglobin, corrected calcium, eGFR, TG, and urine potassium-to-creatinine ratio. Second, because of the self-reporting of medical history, medication, and use of tobacco and alcohol, a social-desirability bias cannot be excluded. It may be responsible for results and conclusions that conflicted with previous research. Furthermore, participants may have forgotten relevant details.

In conclusion, this study demonstrated that a lower sodium diet below 2 g per day was an independent predictor for developing incipient osteoporosis and there was a sex disparity in the association between reduced sodium intake and the risk of incipient osteoporosis. Large population-based prospective epidemiologic studies are warranted to confirm these findings.

## Methods

### Study design and population

Ansan-Anseong cohort data from the Korean Genome and Epidemiology Study (KoGES) were used in this study. Participants for this study were recruited from the fifth follow-up assessment between 2011 and 2012. Prospective data of Ansan-Anseong study are from a medium-sized city (Ansan) and a rural area (Anseong) near Seoul, South Korea to find the potential effect of candidate genetic variation on various chronic illnesses^33^. The comprehensive profile and methods concerning the development of KoGES have been represented previously^33^. Subjects having missing data and those with previously diagnosed osteoporosis, parathyroid-related disease, chronic kidney disease who has eGFR less than 60 or urine albumin-to-creatinine ratio more than 30 or any malignant disease were excluded in this study. All the participants were volunteers and provided written informed consent before enrollment in the study. Their records, except for the date of the survey, were anonymized before analysis. The study protocol was approved by the Institutional Review Board (IRB) of the Hanyang University Seoul Hospital (IRB: HYUH201912015-HE002) and conducted in accordance with the Declaration of Helsinki.

Because our receiver operating characteristic curves analysis demonstrated that e24hUNaE_Tanaka_ had the best precision in estimating the effect of low sodium intake on incipient osteoporosis as compared with Kawasaki, Mage, and INTERSALT methods, the final 3869 participants were divided into quartiles according to their e24hUNaE_Tanaka_ results stratified by sex (Supplemental Figs. 1, 2).

### Anthropometric and clinical measurements

Anthropometric measurements were made by well-trained examiners and followed by standard methods. Participants wore a lightweight gown or underwear. Height was measured to the nearest 0.1 cm and weight was measured to the nearest 0.01 kg using a portable stadiometer with a weighing scale. Body mass index (BMI) was calculated as participants’ weight in kilograms divided by the square of their height in meters.

Blood pressure (BP) was measured twice, using a mercury sphygmomanometer, once in each arm. Participants were allowed to sit for 5-min rest before measuring blood pressure and between the two measurements^34^. The average values of the two recorded systolic and diastolic BPs were used in the analysis.

We analyzed diet and nutritional intake using 24-h recall data provided by KoGES. In the case of KoGES, after the face-to-face survey, a phone survey was conducted. A daily energy intake of less than 400 kcal or more than 4000 kcal was excluded^35^.

### Laboratory tests

Venous blood and urine samples were collected after 8 h overnight fasting and sent to the central laboratory (Seoul Clinical Laboratories, Seoul, Republic of Korea) for quantification by biochemical assays. Blood samples are analyzed using a serum separator tubes (SST) and two ethylenediaminetetraacertic acid tubes(EDTA). Urine sample was analyzed by collecting 10 ml of midstream urine^33^. Fasting plasma concentrations of blood urea nitrogen (BUN), creatinine, albumin, glucose, triglyceride (TG), high-density lipoprotein (HDL)-cholesterol, and low-density lipoprotein (LDL)-cholesterol were determined by an automatic analyzer (ADVIA 1650 and 1680; Siemens, Tarrytown, NY, USA). Glycated hemoglobin (HbA1c) levels were measured by high-performance liquid chromatography (VARIANT II; Bio-Rad Laboratories, Hercules, CA)^36^. Estimated glomerular filtration rate (eGFR) was calculated using the Chronic Kidney Disease Epidemiology Collaboration equation (CKD-EPI)^37^. Urine sodium concentrations were measured using ion-selective electrode and 24-h sodium excretion was estimated based on concentrations of sodium and creatinine in spot urine specimen according to 4 different methods (Supplemental Table 4^38–41^).

### Definition of osteoporosis

The speed-of-sound (SOS, m/s) was used to measure areal BMD (Omnisense 7000 s, Sunlight Medical Ltd, Petah Tivka, Israel). According to World Health Organization Guideline, osteoporosis was defined as a bone mineral density T-score at either distal radius or tibia shaft below −2.5^42^.

### Statistical analysis

All data, including socio-demographic information, medical conditions, anthropometric and clinical measurements, and laboratory results, were presented as mean ± SD or frequencies (and proportions). The normality of the distribution of parameters was analyzed using the Kolmogorov–Smirnov test. If the original data do not follow a Gaussian distribution, the logarithmic transformation was applied to make the distribution more normal. The Kruskal–Wallis test was used to compare quantitative variables and the chi-square test to compare proportions for categorical variables. Linear regression analysis was used to assess the relationship between potential risk factors associated with e24UNaE_Tanaka_ and clinical risk factors of osteoporosis_._ Hazard ratios (HRs) with 95% confidence intervals (CIs) were calculated in multiple Cox-proportional hazards models according to the development of osteoporosis (case vs. control). Kaplan–Meier analysis with the log-rank test was used to compare osteoporosis-free survival between the groups.

Restricted cubic spline (RCS) regression analysis was used to find the possible nonlinear dependency of the association between candidate risk factors and increased risk of the dependent variable^43^.

A two-tailed *P* < 0.05 was considered statistically significant. Statistical Analysis Software version 9.4 (SAS Institute Inc, Cary, NC) was used for all analyses.

## Supplementary Information

Supplementary Information 1.

Supplementary Information 2.

## Data Availability

The data that support the findings of this study are available from the corresponding author, J-S Park, upon reasonable request. Data: Data analyzed in this study were obtained from the Korean Genome and Epidemiology Study 2011–2012 (KoGES; 4851–302), National Research Institute of Health, Centers for Disease Control and Prevention, Ministry for Health and Welfare, Republic of Korea.

## Abbreviations

BMI: Body mass index
BMD: Bone mineral density
BP: Blood pressure
BUN: Blood urea nitrogen
CI: Confidence interval
CKD: Chronic kidney disease
eGFR: Estimated glomerular filtration rate
HbA1c: Glycated hemoglobin A1c
HDL: High density lipoprotein
HR: Hazard ratio
IRB: Institutional Review Board
KoGES: Korean genome and epidemiology study
LDL: Low density lipoprotein
RCS: Restricted cubic spline
SD: Standard deviation
TG: Triglyceride
