# Supplementary material for: The association between dietary sodium intake and osteoporosis
Source: Sci Rep. 2022 Aug 26;12:14594. doi: 10.1038/s41598-022-18830-4 (PMC9418184; doi:10.1038/s41598-022-18830-4)

The Association between Dietary Sodium Intake and Osteoporosis

Susie Hong[1], Jong Wook Choi[2], Joon-Sung Park[1*], and Chang Hwa Lee[1*]

[1]Department of Internal Medicine, Hanyang University College of Medicine, Seoul, Korea

[2]Research Institute of Medical Science, Konkuk University School of Medicine, Chungju, Korea

Supplemental Fig.1

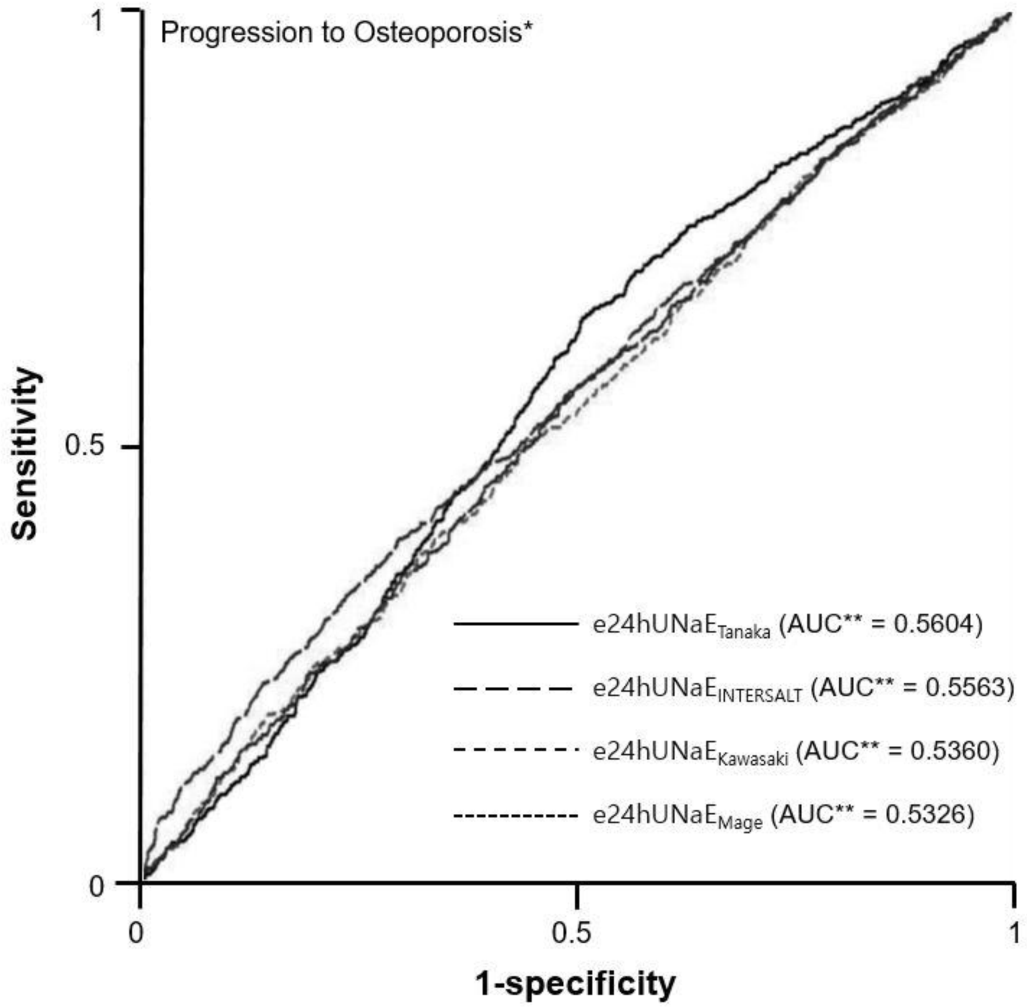

Supplemental Fig.2

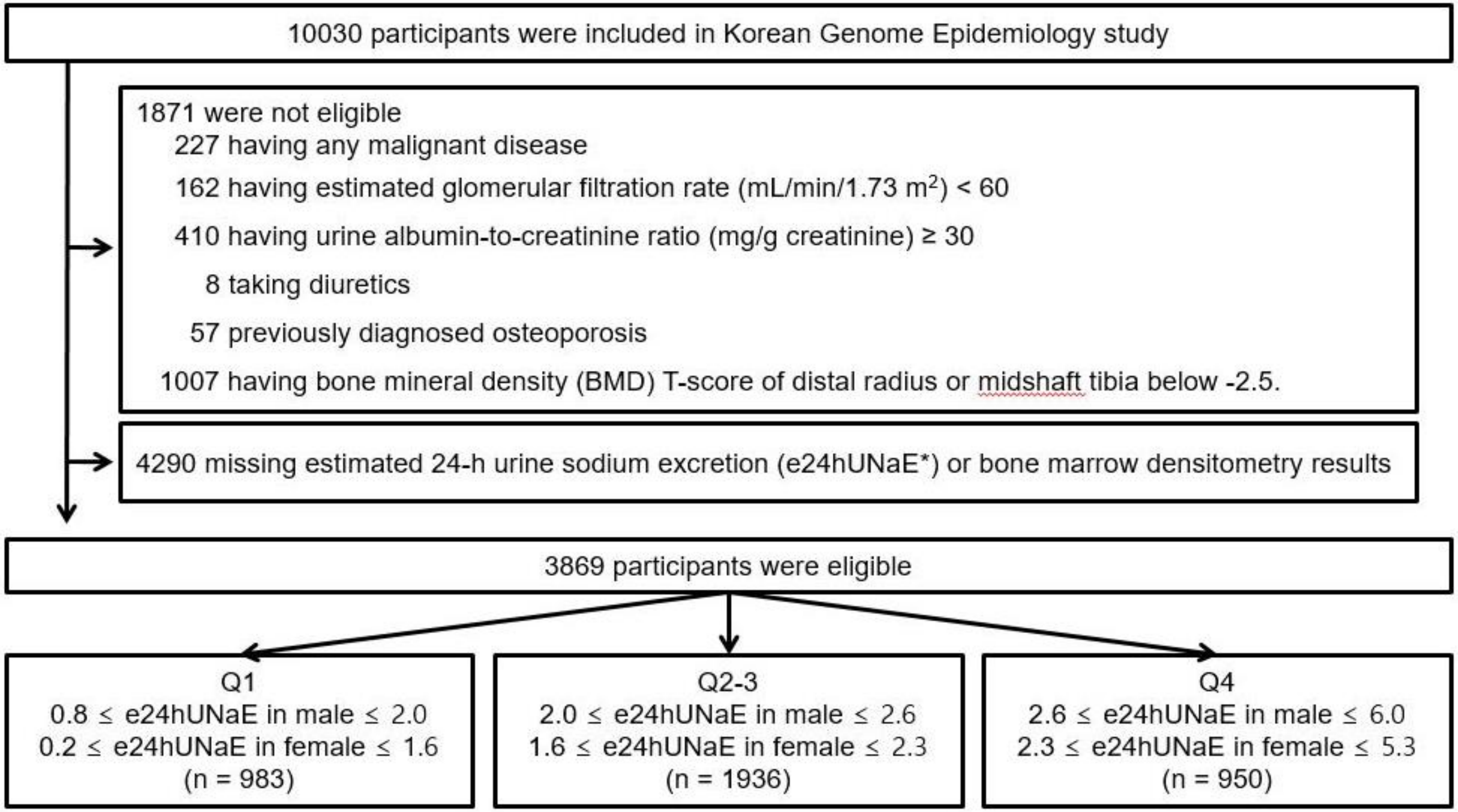

Supplement: Supplementary file 1 — Supplementary Information 1. [file 41598_2022_18830_MOESM1_ESM.pdf]
