# Supplementary material for: The association between dietary sodium intake and osteoporosis
Source: Sci Rep. 2022 Aug 26;12:14594. doi: 10.1038/s41598-022-18830-4 (PMC9418184; doi:10.1038/s41598-022-18830-4)
Supplement: Supplementary file 2 — Supplementary Information 2. [file 41598_2022_18830_MOESM2_ESM.docx]

**The Association between Dietary Sodium Intake and Osteoporosis**

Susie Hong^1^, Jong Wook Choi^2^, Joon-Sung Park^1*^, and Chang Hwa Lee^1*^

^1^Department of Internal Medicine, Hanyang University College of Medicine, Seoul, Korea

^2^Research Institute of Medical Science, Konkuk University School of Medicine, Chungju, Korea

Supplemental Table 1. General characteristics grouped according to e24UNaE_Tanaka_* (g/day)

|  | Male | | |  |  | Female | | | |
| --- | --- | --- | --- | --- | --- | --- | --- | --- | --- |
|  | Quartile 1 | Quartile 2-3 | Quartile 4 |  |  | Quartile 1 | Quartile 2-3 | Quartile 4 |  |
| e24UNaE_Tanaka_ | ≥ 0.8, ≤ 1.9 | > 1.9, ≤ 2.6 | > 2.6, ≤ 5.4 |  |  | ≥ 0.2, ≤ 1.6 | > 1.6, ≤ 2.2 | > 2.2, ≤ 5.3 |  |
| Variables | (n = 473) | (n = 910) | (n = 462) | *P* |  | (n = 510) | (n = 1026) | (n = 488) | *P* |
| Age (year) | 53.7 ± 8.9 | 52.4 ± 8.8 | 51.0 ± 8.6 | <0.0001 |  | 53.3 ± 8.8 | 52.0± 8.8 | 51.7± 8.9 | 0.0053 |
| Current smoker (n, %) | 287 (61) | 479 (53) | 216 (47) | 0.0004 |  | 30 (6) | 30 (3) | 14 (3) | 0.0110 |
| Menopause (n, %) |  |  |  |  |  | 110 (22) | 193 (19) | 89 (18) | 0.7409 |
| Years since menopause (year) |  |  |  |  |  | 11.5 ± 7.8 | 10.6 ± 7.7 | 10.3 ± 7.2 | 0.4849 |
| Body mass index (kg/m^2^) | 23.8 ± 3.2 | 24.3 ± 2.8 | 24.0 ± 3.0 | 0.0097 |  | 24.6 ± 3.2 | 24.6 ± 3.1 | 24.9 ± 3.2 | 0.1448 |
| Waist circumference (cm) | 82.3 ± 8.0 | 84.1 ± 7.5 | 85.0 ± 7.9 | <0.0001 |  | 79.1 ± 9.3 | 81.1 ± 9.5 | 83.0 ± 9.4 | <0.0001 |
| Systolic BP (mmHg) | 122.8 ± 17.0 | 124.8 ± 16.1 | 127.9 ± 17.3 | <0.0001 |  | 120.0 ± 17.5 | 120.0 ± 18.2 | 123.2 ± 18.5 | 0.0075 |
| Diastolic BP (mmHg) | 82.2 ± 10.9 | 84.1 ± 7.5 | 85.0 ± 7.9 | <0.0001 |  | 79.6 ± 9.3 | 81.1 ± 9.5 | 83.0 ± 9.4 | 0.0026 |
| Medical history | | | | | | | | | |
| Diabetes mellitus (n, %) | 22 (5) | 60 (7) | 45 (10) | 0.0077 |  | 23 (5) | 53 (5) | 39 (8) | 0.0336 |
| Hypertension (n, %) | 52 (11) | 112 (12) | 66 (14) | 0.3010 |  | 75 (15) | 134 (13) | 64 (13) | 0.6536 |
| Dyslipidemia (n, %) | 13 (3) | 31 (3) | 16 (3) | 0.7698 |  | 10 (2) | 16 (2) | 8 (2) | 0.8450 |
| Thyroid disease (n, %) | 2 (0) | 8 (1) | 4 (1) | 0.6194 |  | 20 (4) | 58 (6) | 29 (6) | 0.2698 |
| Cardiovascular disease (n, %) | 5 (1) | 12 (1) | 10 (2) | 0.3249 |  | 7 (1) | 17 (2) | 9 (2) | 0.8373 |
| Chronic lung disease (n, %) | 9 (2) | 15 (2) | 2 (0) | 0.1126 |  | 2 (0) | 2 (0) | 4 (1) | 0.1911 |
| Chronic liver disease (n, %) | 27 (6) | 54 (6) | 28 (6) | 0.9713 |  | 19 (4) | 32 (3) | 14 (3) | 0.7290 |
| Laboratory | | | | | | | | | |
| White blood cell (10^9^/L) | 7.1 ± 2.1 | 6.8 ± 1.8 | 6.9 ± 1.8 | 0.0085 |  | 6.5 ± 1.7 | 6.3 ± 1.8 | 6.3 ± 1.7 | 0.0514 |
| Hemoglobin (g/dL) | 14.8 ± 1.1 | 14.8 ± 1.0 | 14.6 ± 1.1 | 0.0030 |  | 12.6 ± 1.1 | 12.6 ± 1.1 | 12.5 ± 1.2 | 0.5402 |
| Platelet (10^3^/μL) | 263.9 ± 65.5 | 256.0 ± 62.7 | 252.4 ± 59.2 | 0.0284 |  | 278.3 ± 63.9 | 266.2 ± 61.9 | 266.1 ± 56.5 | 0.0026 |
| Sodium (mmol/L) | 142.7 ± 2.4 | 142.6 ± 2.3 | 142.3 ± 2.3 | 0.0178 |  | 142.1 ± 2.3 | 142.1 ± 2.3 | 142.0 ± 2.2 | 0.5622 |
| Potassium (mmol/L) | 4.56 ± 0.42 | 4.59 ± 0.42 | 4.57 ± 0.47 | 0.6618 |  | 4.42 ± 0.43 | 4.45 ± 0.41 | 4.46 ± 0.44 | 0.1210 |
| Total protein (g/dL) | 7.19 ± 0.39 | 7.22 ± 0.39 | 7.21 ± 0.44 | 0.3520 |  | 7.20 ± 0.37 | 7.17 ± 0.37 | 7.18 ± 0.38 | 0.3680 |
| Albumin (g/dL) | 4.13 ± 0.21 | 4.13 ± 0.20 | 4.12 ± 0.22 | 0.5471 |  | 4.05 ± 0.18 | 4.04 ± 0.17 | 4.03 ± 0.19 | 0.4945 |
| Corrected calcium (g/dL) | 9.76 ± 0.33 | 9.76 ± 0.33 | 9.78 ± 0.34 | 0.7257 |  | 9.66 ± 0.34 | 9.63 ± 0.34 | 9.67 ± 0.33 | 0.0286 |
| Fasting blood glucose (mg/dL) | 84.5 ± 17.8 | 87.3 ± 22.1 | 87.5 ± 22.7 | 0.0153 |  | 82.5 ± 17.4 | 82.7 ± 15.5 | 87.0 ± 29.9 | 0.2417 |
| Post-prandial glucose (mg/dL) | 119.5 ± 48.6 | 121.7 ± 53.8 | 118.8 ± 53.8 | 0.3115 |  | 127.2 ± 41.7 | 124.7 ± 43.2 | 129.6 ± 55.4 | 0.3564 |
| Hemoglobin A1c (%) | 5.72 ± 0.68 | 5.80 ± 0.88 | 5.85 ± 0.89 | 0.5401 |  | 5.73 ± 0.69 | 5.74 ± 0.87 | 5.88 ± 1.10 | 0.2168 |
| eGFR^†^(mL/min/1.73 m^2^) | 93.2 ± 12.7 | 95.1 ± 12.7 | 98.5 ± 12.8 | <0.0001 |  | 94.5 ± 11.5 | 95.5 ± 11.6 | 95.6 ± 11.7 | 0.1730 |
| Total bilirubin (mg/dL) | 0.65 ± 0.34 | 0.64 ± 0.30 | 0.63 ± 0.37 | 0.1544 |  | 0.52 ± 0.27 | 0.53 ± 0.26 | 0.50 ± 0.24 | 0.2614 |
| AST (IU/L) | 33.5 ± 29.2 | 32.3 ± 16.6 | 32.5 ± 16.4 | 0.9869 |  | 26.9 ± 8.2 | 27.0 ± 12.7 | 27.0 ± 10.2 | 0.2003 |
| ALT (IU/L) | 33.1 ± 31.9 | 33.6 ± 24.5 | 33.2 ± 24.6 | 0.5267 |  | 22.3 ± 10.8 | 22.9 ± 15.7 | 24.1 ± 15.9 | 0.0100 |
| γ- Glutamyl transferase (IU/L) | 48.7 ± 62.7 | 54.2 ± 103.0 | 49.9 ± 60.5 | 0.7782 |  | 18.0 ± 30.6 | 17.9 ± 19.3 | 18.1 ± 17.8 | 0.5069 |
| Triglyceride (mg/dL) | 171.4 ± 98.2 | 179.5 ± 109.0 | 196.7 ± 147.1 | 0.0229 |  | 143.5 ± 68.3 | 142.4 ± 83.6 | 153.4 ± 98.7 | 0.0319 |
| HDL-cholesterol (mg/dL) | 42.3 ± 9.7 | 42.5 ± 10.0 | 42.5 ± 9.7 | 0.9403 |  | 44.4 ± 9.9 | 45.8 ± 9.5 | 44.7 ± 9.2 | 0.0075 |
| LDL-cholesterol (mg/dL) | 107.0 ± 29.6 | 107.4 ± 30.0 | 99.8 ± 31.1 | <0.0001 |  | 111.1 ± 28.5 | 110.5 ± 29.6 | 109.7 ± 30.9 | 0.4758 |
| C-reactive protein (mg/dL) | 0.27 ± 0.46 | 0.21 ± 0.29 | 0.21 ± 0.51 | 0.0191 |  | 0.23 ± 0.51 | 0.18 ± 0.24 | 0.19 ± 0.29 | 0.0189 |
| UACR (mg/g Cr) | 7.9 ± 5.7 | 8.9 ± 5.7 | 10.7 ± 7.0 | <0.0001 |  | 10.7 ± 7.0 | 11.2 ± 6.7 | 13.8 ± 7.9 | <0.0001 |
| Daily intake | | | | | | | | | |
| Dietary energy intake (Kcal/day) | 2032 ± 760 | 2112 ± 834 | 2153 ± 743 | 0.0357 |  | 1912 ± 818 | 1984 ± 777 | 2072 ± 899 | <0.0001 |
| Dietary Na intake (g/day) | 3.41 ± 1.92 | 3.55 ± 1.91 | 3.62 ± 1.77 | 0.0361 |  | 3.01 ± 1.59 | 3.24 ± 1.85 | 3.32 ± 1.73 | 0.0213 |
| Dietary K intake (g/day) | 2.69 ± 1.37 | 2.78 ± 1.37 | 2.77 ± 1.18 | 0.2157 |  | 2.58 ± 1.34 | 2.79 ± 1.44 | 2.85 ± 1.50 | 0.0070 |
| Dietary Ca intake (g/day) | 0.48 ± 0.28 | 0.51 ± 0.30 | 0.52 ± 0.28 | 0.0125 |  | 0.48 ± 0.27 | 0.52 ± 0.31 | 0.53 ± 0.30 | 0.0826 |
| Daily alcohol intake (g/day) | 22.7 ± 26.9 | 26.2 ± 30.5 | 30.1 ± 36.6 | 0.0037 |  | 5.4 ± 17.1 | 4.8 ± 8.5 | 6.7 ± 13.3 | 0.6149 |
| Fasting morning urine sample | | | | | | | | | |
| e24UNaE_Tanaka_* (g/day) | 1.69 ± 0.23 | 2.28 ± 0.18 | 2.99 ± 0.38 | <0.0001 |  | 1.24 ± 0.26 | 1.89 ± 0.20 | 2.58 ± 0.33 | <0.0001 |
| FE_Na_ (%) | 0.5 ± 0.2 | 0.8 ± 0.3 | 1.4 ± 0.7 | <0.0001 |  | 0.6 ± 0.4 | 0.8 ± 0.4 | 1.4 ± 0.8 | <0.0001 |
| UKCR (mmol/mmol) | 4.3 ± 2.3 | 4.9 ± 2.5 | 7.3 ± 7.3 | <0.0001 |  | 6.2 ± 3.9 | 6.6 ± 3.5 | 8.7 ± 6.3 | <0.0001 |
| UNaKR (mmol/mmol) | 2.3 ± 1.3 | 3.5 ± 1.7 | 4.5 ± 2.2 | <0.0001 |  | 2.4 ± 1.4 | 3.3 ± 1.6 | 4.2 ± 1.7 | <0.0001 |
| UCaCR (mg/dL/mg/dL) | 0.08 ± 0.06 | 0.11± 0.07 | 0.14 ± 0.08 | <0.0001 |  | 0.12 ± 0.08 | 0.16 ± 0.09 | 0.78 ± 13.27 | <0.0001 |
| FE_Ca_ (%) | 0.8 ± 0.5 | 1.0 ± 0.6 | 1.2 ± 0.7 | <0.0001 |  | 0.9 ± 0.6 | 1.1 ± 0.7 | 5.4 ± 90.2 | <0.0001 |
| Bone mineral densitometry | | | | | | | | | |
| SOS of DR at base (m/s) | 4187 ± 139 | 4188 ± 145 | 4199 ± 147 | 0.6235 |  | 4247 ± 173 | 4262 ± 164 | 4229 ± 156 | 0.0024 |
| ΔSOS of DR (m/s/year) | -0.72 ± 1.29 | -0.63 ± 1.20 | -0.73 ± 1.20 | 0.2410 |  | -1.06 ± 1.22 | -0.90 ± 1.21 | -0.86 ± 1.17 | 0.0024 |
| T-score of DR at base | 0.50 ± 1.13 | 0.51 ± 1.17 | 0.61 ± 1.21 | 0.5680 |  | 0.76 ± 1.44 | 0.72 ± 1.37 | 0.43 ± 1.30 | 0.0021 |
| ΔT-score of DR (/year) | -0.23 ± 0.43 | -0.20 ± 0.40 | -0.24 ± 0.42 | 0.2404 |  | -0.37 ± 0.44 | -0.32 ± 0.45 | -0.29 ± 0.41 | 0.0751 |
| SOS of MT at base (m/s) | 3987 ± 149 | 4005 ± 116 | 3995 ± 109 | 0.1988 |  | 3917 ± 146 | 3931 ± 137 | 3922 ± 131 | 0.0556 |
| ΔSOS of MT (m/s/year) | -0.58 ± 2.69 | -0.69 ± 1.04 | -0.57 ± 0.95 | 0.1824 |  | -0.98 ± 1.29 | -0.93 ± 1.37 | -0.89 ± 1.31 | 0.6502 |
| T-score of MT at base | 0.35 ± 1.17 | 0.46 ± 1.13 | 0.37 ± 1.08 | 0.3138 |  | -0.31 ± 1.34 | -0.17 ± 1.25 | -0.25 ± 1.20 | 0.0048 |
| ΔT-score of MT (/year) | -0.28 ± 0.43 | -0.27 ± 0.43 | -0.22 ± 0.37 | 0.1738 |  | -0.33 ± 0.47 | -0.31 ± 0.49 | -0.30 ± 0.48 | 0.6925 |
| Osteoporosis progression^ǂ^ (n, %) | 66 (14) | 137 (15) | 69 (15) | 0.8527 |  | 160 (31) | 267 (26) | 135 (28) | 0.0879 |

Results are expressed as mean ± SD or frequencies (and proportions).

e24UNaE, estimated 24-h urine sodium excretion; BP, blood pressure; eGFR, estimated glomerular filtration rate; AST, Aspartate aminotransferase; ALT, Alanine aminotransferase; HDL, high-density lipoprotein; LDL, low-density lipoprotein; UACR, Urine albumin/Cr ratio; Cr, creatinine, Na, sodium; K, potassium; Ca, calcium; FE_Na_, fractional excretion of sodium; UKCR, urine potassium/creatinine ratio; UNaKR, urine sodium/potassium ratio; FE_Ca_, fractional excretion of calcium; SoS, speed of sound; DR, distal radius; MT, midshaft tibia.

*e24UNaE calculated using Tanaka method.

^†^estimated using the Chronic Kidney Disease Epidemiology Collaboration equation.

^ǂ^ defined as a bone mineral density T-score at ether distal radius or midshaft of tibia below -2.5.

Supplemental Table 2. Linear regression for e24UNaE_Tanaka_ (g/day)

|  | Male | | | | | | |  | Female | | | | |
| --- | --- | --- | --- | --- | --- | --- | --- | --- | --- | --- | --- | --- | --- |
|  | Crude | |  | Model I | | | |  | Crude | |  | Model I | |
| Variable | Slope | *P* |  | Slope | | *P* | |  | Slope | *P* |  | Slope | *P* |
| Age (year) | -0.0064 | <0.0001 |  | |  | |  |  | -0.0051 | <0.0001 |  |  |  |
| Current smoker (vs. non-smoker) | -0.0592 | 0.0002 |  | |  | |  |  | -0.0783 | 0.0116 |  |  |  |
| Menopause (vs. pre-menopause) |  |  |  | |  | |  |  | -0.0303 | 0.5103 |  |  |  |
| Years since menopause (year) |  |  |  | |  | |  |  | -0.0055 | 0.0902 |  |  |  |
| Body mass index (kg/m^2^) | 0.0002 | 0.9631 |  | |  | |  |  | 0.0054 | 0.1559 |  |  |  |
| Waist circumference (cm) | 0.0077 | <0.0001 |  | | 0.0074 | | <0.0001 |  | 0.0077 | <0.0001 |  | 0.0075 | <0.0001 |
| Systolic BP (mmHg) | 0.0032 | <0.0001 |  | | 0.0032 | | <0.0001 |  | 0.0011 | 0.1052 |  |  |  |
| Diastolic BP (mmHg) | 0.0041 | 0.0004 |  | | 0.0041 | | 0.0004 |  | 0.0010 | 0.3274 |  |  |  |
| Laboratory | | | | | | | | | | | | | |
| White blood cell (10^9^/L) | -0.0193 | 0.0034 |  | | -0.0135 | | 0.0474 |  | -0.0096 | 0.1594 |  |  |  |
| Hemoglobin (g/dL) | -0.0450 | 0.0001 |  | | -0.0429 | | 0.0002 |  | -0.0131 | 0.2090 |  |  |  |
| Platelet (10^3^/μL) | -0.0007 | 0.0004 |  | | -0.0006 | | 0.0015 |  | -0.0006 | 0.0042 |  | -0.0005 | 0.0058 |
| Sodium (mmol/L) | -0.0151 | 0.0055 |  | | -0.0146 | | 0.0072 |  | -0.0038 | 0.4632 |  |  |  |
| Potassium (mmol/L) | 0.0118 | 0.6823 |  | |  | |  |  | 0.0375 | 0.1881 |  |  |  |
| Total protein (g/dL) | 0.0065 | 0.8349 |  | |  | |  |  | -0.0282 | 0.3831 |  |  |  |
| Albumin (g/dL) | -0.0987 | 0.1050 |  | |  | |  |  | ~~-~~0.0568 | 0.3992 |  |  |  |
| Corrected calcium (g/dL) | 0.0521 | 0.1679 |  | |  | |  |  | 0.0301 | 0.3979 |  |  |  |
| Fasting blood glucose (mg/dL) | 0.0012 | 0.0483 |  | | 0.0011 | | 0.0610 |  | 0.0021 | 0.0003 |  | 0.0020 | 0.0006 |
| Post-prandial glucose (mg/dL) | -0.0001 | 0.6499 |  | |  | |  |  | -0.0002 | 0.5409 |  |  |  |
| Hemoglobin A1c (%) | 0.0363 | 0.0152 |  | | 0.0335 | | 0.0257 |  | 0.0405 | 0.0026 |  | 0.0376 | 0.0052 |
| eGFR* (mL/min/1.73 m^2^) | 0.0068 | <0.0001 |  | | 0.0061 | | <0.0001 |  | 0.0024 | 0.0223 |  | 0.0008 | 0.4989 |
| Total bilirubin (mg/dL) | -0.0592 | 0.1229 |  | |  | |  |  | -0.0461 | 0.3233 |  |  |  |
| AST (IU/L) | -0.0004 | 0.4699 |  | |  | |  |  | 0.0002 | 0.8746 |  |  |  |
| ALT (IU/L) | -0.0002 | 0.6488 |  | |  | |  |  | 0.0020 | 0.0215 |  | 0.0017 | 0.0365 |
| γ- Glutamyl transferase (IU/L) | -0.0001 | 0.8025 |  | |  | |  |  | 0.0004 | 0.4567 |  |  |  |
| Triglyceride (mg/dL) | 0.0003 | 0.0055 |  | | 0.0003 | | 0.0116 |  | 0.0003 | 0.0257 |  | 0.0003 | 0.0261 |
| HDL-cholesterol (mg/dL) | -0.0009 | 0.4937 |  | |  | |  |  | -0.0004 | 0.7749 |  |  |  |
| LDL-cholesterol (mg/dL) | -0.0020 | <0.0001 |  | | -0.0019 | | <0.0001 |  | -0.0004 | 0.2730 |  |  |  |
| C-reactive protein (mg/dL) | -0.0748 | 0.0159 |  | | -0.0731 | | 0.0189 |  | -0.0776 | 0.0271 |  | -0.0762 | 0.0315 |
| UACR (mg/g Cr) | 0.0125 | <0.0001 |  | | 0.0123 | | <0.0001 |  | 0.0109 | <0.0001 |  | 0.0108 | <0.0001 |
| Daily intake | | | | | | | | | | | | | |
| Dietary energy intake (Kcal/day) | 0.0001 | 0.0014 |  | | 0.0001 | | 0.0028 |  | 0.0001 | 0.1151 |  |  |  |
| Dietary Na intake (g/day) | 0.0001 | 0.0011 |  | |  | |  |  |  |  |  |  |  |
| Dietary K intake (g/day) | 0.0001 | 0.0726 |  | |  | |  |  |  |  |  |  |  |
| Dietary Ca intake (g/day) | 0.0001 | 0.0056 |  | |  | |  |  |  |  |  |  |  |
| Daily alcohol intake (g/day) | 0.0013 | 0.0096 |  | |  | |  |  |  |  |  |  |  |
| Fasting morning urine sample | | | | | | | | | | | | | |
| FE_Na_ (%) | 0.7884 | <0.0001 |  | | 0.7860 | | <0.0001 |  | 0.5599 | <0.0001 |  | 0.5522 | <0.0001 |
| UKCR (mmol/mmol) | 0.0505 | <0.0001 |  | | 0.0500 | | <0.0001 |  | 0.0348 | <0.0001 |  | 0.0335 | <0.0001 |
| UNaKR (mmol/mmol) | 0.1204 | <0.0001 |  | | 0.1190 | | <0.0001 |  | 0.1254 | <0.0001 |  | 0.1236 | <0.0001 |
| UCaCR (mg/dL/mg/dL) | 2.1487 | <0.0001 |  | | 2.1308 | | <0.0001 |  | 0.0019 | 0.3052 |  |  |  |
| FE_Ca_ (%) | 0.2268 | <0.0001 |  | | 0.2205 | | <0.0001 |  | 0.0003 | 0.2986 |  |  |  |
| Bone mineral densitometry | | | | | | | | | | | | | |
| SOS of DR at base (m/s) | 0.0001 | 0.3925 |  | |  | |  |  | 0.0002 | 0.0094 |  | 0.0002 | 0.0167 |
| ΔSOS of DR (m/s/year) | 0.0030 | 0.8069 |  | |  | |  |  | 0.0183 | 0.1465 |  |  |  |
| T-score of DR at base | -0.0107 | 0.3437 |  | |  | |  |  | -0.0254 | 0.0071 |  | -0.0236 | 0.0129 |
| ΔT-score of DR (/year) | 0.0053 | 0.8847 |  | |  | |  |  | 0.0553 | 0.1268 |  |  |  |
| SOS of MT at base (m/s) | 0.0001 | 0.4294 |  | |  | |  |  | 0.0001 | 0.1243 |  |  |  |
| ΔSOS of MT (m/s/year) | 0.0012 | 0.8878 |  | |  | |  |  | 0.0159 | 0.1621 |  |  |  |
| T-score of MT at base | 0.0027 | 0.8154 |  | |  | |  |  | 0.0169 | 0.0986 |  |  |  |
| ΔT-score of MT (/year) | 0.0581 | 0.1079 |  | |  | |  |  | 0.0378 | 0.2305 |  |  |  |

Model I, adjusted for age and smoking history.

Supplemental Table 3. Subgroup analysis of Cox proportional-hazard model for osteoporosis*

|  | Male | | | | |  | Female | | | | | |
| --- | --- | --- | --- | --- | --- | --- | --- | --- | --- | --- | --- | --- |
|  | Model I | |  | Model II | |  | Model I | |  | | Model II | |
| Variable | OR | 95% CI |  | OR | 95% CI |  | OR | 95% CI |  | OR | | 95% CI |
| Menopause (vs. pre-menopause) |  |  |  |  |  |  | 1.040 | 0.828-1.305 |  |  | |  |
| Years since menopause (year) |  |  |  |  |  |  | 1.001 | 0.979-1.025 |  |  | |  |
| Body mass index (kg/m^2^) | 1.024 | 0.992-1.056 |  |  |  |  | 1.045 | 1.025-1.065 |  |  | |  |
| Waist circumference (cm) | 1.002 | 0.990-1.014 |  |  |  |  | 1.024 | 1.017-1.031 |  |  | |  |
| Systolic BP (mmHg) | 1.002 | 0.997-1.008 |  |  |  |  | 1.010 | 1.007-1.013 |  |  | |  |
| Diastolic BP (mmHg) | 1.002 | 0.993-1.011 |  |  |  |  | 1.013 | 1.007-1.018 |  |  | |  |
| Medical history | | | | | | | | | | | | |
| Diabetes mellitus | 1.053 | 0.753-1.473 |  |  |  |  | 1.111 | 0.864-1.494 |  |  | |  |
| Hypertension | 1.028 | 0.786-1.344 |  |  |  |  | 1.351 | 1.157-1.577 |  | 1.156 | | 0.894-1.494 |
| Dyslipidemia | 1.044 | 0.896-1.218 |  |  |  |  | 1.093 | 0.676-1.767 |  |  | |  |
| Thyroid disease | 2.242 | 0.834-6.024 |  |  |  |  | 1.238 | 0.914-1.675 |  |  | |  |
| Cardiovascular disease | 1.038 | 0.569-1.894 |  |  |  |  | 1.296 | 0.849-1.978 |  |  | |  |
| Chronic lung disease | 1.048 | 0.717-2.926 |  |  |  |  | 2.954 | 1.086-8.033 |  | 2.641 | | 1.415-4.927 |
| Chronic liver disease | 1.362 | 0.959-1.934 |  |  |  |  | 1.299 | 0.859-1.965 |  |  | |  |
| Laboratory | | | | | | | | | | | | |
| White blood cell (10^9^/L) | 1.005 | 0.955-1.059 |  |  |  |  | 1.004 | 0.969-1.040 |  |  | |  |
| Hemoglobin (g/dL) | 1.009 | 0.926-1.099 |  |  |  |  | 1.064 | 1.008-1.122 |  |  | |  |
| Platelet (10^3^/μL) | 1.001 | 0.999-1.002 |  |  |  |  | 1.000 | 0.999-1.001 |  |  | |  |
| Sodium (mmol/L) | 1.008 | 0.967-1.052 |  |  |  |  | 1.050 | 1.021-1.081 |  |  | |  |
| Potassium (mmol/L) | 1.038 | 0.821-1.312 |  |  |  |  |  | 0.824-1.111 |  |  | |  |
| Total protein (g/dL) | 1.184 | 0.939-1.494 |  |  |  |  | 0.792 | 0.678-0.924 |  | 0.721 | | 0.549-0.946 |
| Albumin (g/dL) | 1.177 | 0.808-1.713 |  |  |  |  | 0.698 | 0.542-0.898 |  | 0.637 | | 0.365-1.114 |
| Corrected calcium (g/dL) | 1.070 | 0.853-1.343 |  |  |  |  | 1.333 | 1.159-1.533 |  |  | |  |
| Fasting blood glucose (mg/dL) | 1.000 | 0.995-1.004 |  |  |  |  | 0.996 | 0.992-1.001 |  |  | |  |
| Post-prandial glucose (mg/dL) | 1.000 | 0.999-1.002 |  |  |  |  | 1.000 | 0.998-1.004 |  |  | |  |
| Hemoglobin A1c (%) | 1.035 | 0.952-1.125 |  |  |  |  | 1.089 | 1.024-1.159 |  |  | |  |
| eGFR* (mL/min/1.73 m^2^) | 1.006 | 0.998-1.015 |  |  |  |  | 1.003 | 0.997-1.008 |  |  | |  |
| Total bilirubin (mg/dL) | 1.122 | 0.807-1.560 |  |  |  |  | 0.697 | 0.678-0.924 |  | 0.721 | | 0.549-0.946 |
| AST (IU/L) | 1.000 | 0.994-1.005 |  |  |  |  |  |  |  |  | |  |
| ALT (IU/L) | 1.001 | 0.998-1.004 |  |  |  |  | 0.999 | 0.995-1.003 |  |  | |  |
| γ- Glutamyl transferase (IU/L) | 1.000 | 0.999-1.001 |  |  |  |  | 1.001 | 0.998-1.004 |  |  | |  |
| Triglyceride (mg/dL) | 1.000 | 0.999-1.001 |  |  |  |  | 1.001 | 1.001-1.002 |  |  | |  |
| HDL-cholesterol (mg/dL) | 1.000 | 0.991-1.010 |  |  |  |  | 0.998 | 0.992-1.004 |  |  | |  |
| LDL-cholesterol (mg/dL) | 1.002 | 0.999-1.005 |  |  |  |  | 1.003 | 1.001-1.005 |  |  | |  |
| C-reactive protein (mg/dL) | 1.221 | 1.006-1.482 |  | 1.354 | 1.076-1.705 |  | 1.033 | 0.957-1.116 |  |  | |  |
| UACR (mg/g Cr) | 1.041 | 1.022-1.060 |  | 1.043 | 1.024-1.063 |  | 1.008 | 0.997-1.019 |  |  | |  |
| Daily intake | | | | | | | | | | | | |
| Dietary Na intake (g/day) | 1.000 | 0.999-1.001 |  |  |  |  | 1.000 | 0.999-1.001 |  |  | |  |
| Dietary K intake (g/day) | 1.000 | 0.999-1.001 |  |  |  |  | 1.000 | 0.999-1.001 |  |  | |  |
| Dietary Ca intake (g/day) | 1.000 | 0.999-1.001 |  |  |  |  | 1.000 | 0.999-1.001 |  |  | |  |
| Daily alcohol intake (g/day) | 0.998 | 0.994-1.002 |  |  |  |  | 1.007 | 0.995-1.020 |  |  | |  |
| Fasting morning urine sample | | | | | | | | | | | | |
| e24UNaE_Tanaka_ (g/day) | 0.858 | 0.675-1.091 |  |  |  |  | 0.851 | 0.724-1.001 |  |  | |  |
| FE_Na_ (%) | 0.954 | 0.759-1.200 |  |  |  |  | 0.864 | 0.767-0.973 |  | 0.947 | | 0.813-1.103 |
| UKCR (mmol/mmol) | 1.013 | 0.912-1.039 |  |  |  |  | 1.028 | 1.015-1.041 |  | 1.023 | | 1.008-1.038 |
| UNaKR (mmol/mmol) | 0.974 | 0.912-1.039 |  |  |  |  | 0.953 | 0.906-1.002 |  |  | |  |
| UCaCR (mg/dL/mg/dL) | 1.879 | 0.363-9.709 |  |  |  |  | 1.010 | 0.960-1.063 |  |  | |  |
| FE_Ca_ (%) | 1.115 | 0.913-1.361 |  |  |  |  | 1.001 | 0.994-1.009 |  |  | |  |

*defined as a bone mineral density T-score at distal radius or tibia shaft below -2.5.

Model I, performed using age, sex, and smoking history as covariates

Model II, performed using age, sex, and smoking history as covariates and body mass index, waist circumference, systolic BP, diastolic BP, hemoglobin, sodium, corrected calcium, hemoglobin A1c, triglyceride, LDL-cholesterol, and UACR as predictors.

HR, hazard ratio; CI, confidence interval.

Supplemental Table 4. Four methods for estimated 24-h urine sodium excretion (e24UNaE, mg/day)

| Method | Formula | Reference |
| --- | --- | --- |
| Kawasaki | 23 x 16.3 x (Na_spot_ (mmol/L) / Cr_spot_ (mmol/L) x PrUCr24h)^0.5^. | (38-40) |
|  | PrUCr24h (mg/day) for male = 15.12 x Wt (kg) + 7.39 x Ht (cm) - 12.63 x age (y) - 79.9. |  |
|  | PrUCr24h (mg/day) for female = 8.58 x Wt (kg) + 5.09 x Ht (cm) - 4.72 x age (y) - 79.95. |  |
| Tanaka | 23 x 21.98 x (Na_spot_ / Cr_spot_ x PrUCr24h)^0.392^. | (38,39) |
|  | PrUCr24h (mg/day) = 14.89 x Wt (kg) + 16.14 x Ht (cm) - 2.04 x age (y) - 2244.45. |  |
| Mage | 23 x (Na_spot_ (mmol/L) / Cr_spot_ (mmol/L) x PrUCr24h). | (40) |
|  | PrUCr24h (mg/day) for male = 0.00179 x [140 - age (y)] x [Wt^1.5^ (kg) x Ht^0.5^ (cm)] x {1 + 0.18 x A* x [1.366 - 0.0159 x BMI(kg/m^2^)]}. |  |
|  | PrUCr24h (mg/day) for female = 0.00163 x [140 - age (y)] x [Wt^1.5^ (kg) x Ht^0.5^ (cm)] x {1 + 0.18 x A* x [1.429 - 0.0198 x BMI (kg/m^2^)]}. |  |
| INTERSALT | 23 x [25.46 + 0.46 x Na_spot_ (mmol/L) - 2.75 x Cr_spot_ (mmol/L) - 0.13 x K_spot_ (mmol/L) + 4.10 x BMI(kg/m^2^) - 0.26 x age (y)] for male | (38, 41) |
|  | 23 x [5.07 + 0.34 x Na_spot_ (mmol/L) - 2.16 x Cr_spot_ (mmol/L) - 0.09 x K_spot_ (mmol/L) + 2.39 x BMI(kg/m^2^) - 0.26 x age (y) - 0.03 x age^2^ (y)] for female |  |

*A of African-American of black is 1, and other = 0.

Na_spot_, spot urinary sodium; K_spot_, spot urinary potassium; Cr_spot_, spot urinary creatinine; Wt, weight; Ht, height; PrUCr24h, predicted 24-h urinary creatinine; BMI, body mass index.

**Supplemental Fig 1. ROC curves representing the prediction capacity of risk for progression to osteoporosis*.**

As compared with e24hUNaE_Tanaka_ (AUC****** = 0.5604, 95% CI****** = 0.5373-0.5836), e24hUNaE_INTERSALT_ (AUC****** = 0.5563, 95% CI****** = 0.5317-0.5808, *P******** = 0.6850), e24hUNaE_Kawasaki_ (AUC****** = 0.5360, 95% CI****** = 0.5121-0.5599, *P******** 0.3858), and e24hUNaE_Mage_ (AUC****** = 0.5326, 95% CI****** = 0.5086-0.5566, *P******** = 0.3134) have poor precision in predicting risk of osteoporosis progression. However, there is significant difference between them.

*Defined as an eGFR less than 60 mL/min/1.73 m2 and UACR of 30 mg/g Cr or more.

ROC, Receiver-operating characteristic; AUC, areas under the ROC curves; CI, confidence interval.

******Calculated by logistic regression analysis using age, sex, and smoking history as covariates and body mass index, waist circumference, systolic BP, diastolic BP, hemoglobin, sodium, corrected calcium, hemoglobin A1c, triglyceride, LDL-cholesterol, UACR, and urine potassium/creatinine ratio as predictors.

***Estimated using nonparametric methods previously described by DeLong, et. al.

e24hUNaE_Tanaka_, Tanaka method for estimating 24-h urinary sodium excretion; e24hUNaE_INTERSALT_, INTERSALT method for estimating 24-h urinary sodium excretion; e24hUNaE_Kawasaki_, Kawasaki method for estimating 24-h urinary sodium excretion; e24hUNaE_Mage_, Mage method for estimating 24-h urinary sodium excretion.

**Supplemental Fig 2. Flow chart of the study group enrollment process.**

Q, e24UNaE_Tanaka_ quintile
